# Advance distribution of misoprostol for prevention of postpartum hemorrhage (PPH) at home births in two districts of Liberia

**DOI:** 10.1186/1471-2393-14-189

**Published:** 2014-06-04

**Authors:** Jeffrey Michael Smith, Saye Dahn Baawo, Marion Subah, Varwo Sirtor-Gbassie, Cuallau Jabbeh Howe, Gbenga Ishola, Bentoe Z Tehoungue, Vikas Dwivedi

**Affiliations:** 1 MCHIP, Jhpiego, 1776 Massachusetts Ave., NW#300, Washington, DC 20036, USA; 2 Ministry of Health and Social Welfare, Monrovia, Republic of Liberia; 3 Jhpiego/Liberia, Monrovia, Republic of Liberia; 4 MCHIP/Liberia, Monrovia, Republic of Liberia; 5 Jhpiego/Nigeria, Abuja, Republic of Liberia; 6 MCHIP/JSI Research & Training, Inc, Boston, MA 02210, USA

**Keywords:** Advance distribution, Misoprostol, Postpartum hemorrhage, Uterotonic, Coverage, CHW, AMTSL, Home birth, Liberia

## Abstract

**Background:**

A postpartum hemorrhage prevention program to increase uterotonic coverage for home and facility births was introduced in two districts of Liberia. Advance distribution of misoprostol was offered during antenatal care (ANC) and home visits. Feasibility, acceptability, effectiveness of distribution mechanisms and uterotonic coverage were evaluated.

**Methods:**

Eight facilities were strengthened to provide PPH prevention with oxytocin, PPH management and advance distribution of misoprostol during ANC. Trained traditional midwives (TTMs) as volunteer community health workers (CHWs) provided education to pregnant women, and district reproductive health supervisors (DRHSs) distributed misoprostol during home visits. Data were collected through facility and DRHS registers. Postpartum interviews were conducted with a sample of 550 women who received advance distribution of misoprostol on place of delivery, knowledge, misoprostol use, and satisfaction.

**Results:**

There were 1826 estimated deliveries during the seven-month implementation period. A total of 980 women (53.7%) were enrolled and provided misoprostol, primarily through ANC (78.2%). Uterotonic coverage rate of all deliveries was 53.5%, based on 97.7% oxytocin use at recorded facility vaginal births and 24.9% misoprostol use at home births. Among 550 women interviewed postpartum, 87.7% of those who received misoprostol and had a home birth took the drug. Sixty-three percent (63.0%) took it at the correct time, and 54.0% experienced at least one minor side effect. No serious adverse events reported among enrolled women. Facility-based deliveries appeared to increase during the program.

**Conclusions:**

The program was moderately effective at achieving high uterotonic coverage of all births. Coverage of home births was low despite the use of two channels of advance distribution of misoprostol. Although ANC reached a greater proportion of women in late pregnancy than home visits, 46.3% of expected deliveries did not receive education or advance distribution of misoprostol. A revised community-based strategy is needed to increase advance distribution rates and misoprostol coverage rates for home births. Misoprostol for PPH prevention appears acceptable to women in Liberia. Correct timing of misoprostol self-administration needs improved emphasis during counseling and education.

## Background

Maternal mortality remains unacceptably high in Liberia, at 770 maternal deaths per 100,000 live births^a^[1]. Postpartum hemorrhage (PPH) is the leading cause of maternal mortality, accounting for approximately 42% of deaths in Liberia and 34% in Africa overall [2]. In Liberia the health system’s capacity to provide emergency obstetric care is limited by the numbers of surgeons, physicians and midwives, poor geographic access, a weak referral system and inadequate infrastructure [3]. Most (95%) of public health facilities were destroyed by the war [4]. Post-conflict Liberia has made significant strides in rebuilding its health system. In 2009 a Basic Package of Health Services was introduced, which includes key maternal and child health interventions. Despite these efforts, one-third (34.0%) of pregnant women do not receive the recommended four or more antenatal care (ANC) visits [5]. Deliveries in a health facility have increased from 36.9% in 2007 to 55.8% in 2013 [6]. Still, 38.9% of women in Liberia give birth without the assistance of a skilled birth attendant [6].

World Health Organization recommendations for the prevention of PPH emphasize the provision of a uterotonic to all women during the third stage of labor [7]. Oxytocin is the preferred uterotonic for prevention of PPH. Misoprostol however is a reasonable alternative, especially in home birth settings where a qualified provider or injectable oxytocin are unavailable. Misoprostol is a uterotonic used for the prevention and treatment of PPH [8]. It is manufactured in tablet form and is taken orally for PPH prevention (three 200 mcg tablets, total dose 600 mcg) [9]. It is inexpensive, easy to store and has an excellent safety profile [10]. Various studies have demonstrated misoprostol’s effectiveness in preventing PPH, reducing the need for additional interventions and reducing the need for referrals in a variety of community-based settings [11]. A number of community-based programs for PPH prevention at home birth using misoprostol have been safely conducted with a variety of cadres involved in drug distribution [12]. Advocates are calling for a scale up of this approach [13].

It is recognized that the distribution of misoprostol through community health workers (CHWs) for PPH prevention could be a significant step in reducing maternal deaths in low-resource settings [14]. This article contributes evidence concerning the safety and effectiveness of advance distribution for self-administration of misoprostol immediately after birth for PPH prevention, in settings where oxytocin use is not feasible.

## Methods

### Study setting

To reduce mortality from PPH the Liberia Ministry of Health and Social Welfare (MOHSW) is implementing two concurrent strategies to increase uterotonic coverage. The first aims to increase use of active management of the third stage of labor (AMTSL) at institutional deliveries, with a focus on the provision of oxytocin as part of active management. The second is to study counseling of pregnant women either during ANC or home visits by a health worker and the advance distribution of misoprostol to those women late in pregnancy for use at home births.

Both interventions were implemented simultaneously in two districts of Grand Bassa County in 2012 and 2013. District #3 and Campwood districts were selected for this evaluation based on Ministry of Health and Social Welfare (MOHSW) priorities, compelling public health need, interest of the district leadership and existing program funding support from USAID. Because pregnant women from Campwood district often seek services in Fenutoli of neighboring Bong County and vice versa, the Fenutoli population and clinic were included in the Campwood parameters in Table 1.

**Table 1:** Population and maternal health parameters for district #3 and Campwood District, Liberia

	**District #3**	**Campwood**	**Total**
Total population in each district^a^	51,167	18,397	69,564
Estimated number of pregnant women in a year^b^	2,302	828	3,130
% ANC coverage – ANC 1			96.0%^c^
% ANC coverage – ANC 4			66.0%^d^
% facility based births			55.8%^c^
Total number of villages in each district	110	79	189
Total # and types of facilities in each district	1 hospital 6 clinics	2 clinics	9
Number and type of health workers in the districts	CMs (3), RN/CM (1) RNs (12), PAs (2), DRHS (2)		
Trained Traditional Midwives	110	50	

^a^2011 population projection (MOHSW).

^b^based on the crude birth rate of 45.0 (MOHSW).

^c^National level (LDHS 2013).

^d^National level (LDHS 2007) 2013 data not yet available.

### Protection of human subjects

This project was approved by the Institutional Review Board at the Johns Hopkins Bloomberg School of Public Health, U.S.A (IRB00004451) and the Liberian Institute for Biomedical Research/National Health Science Research Ethics Committee (ref EC/LIBR/012/021) in October 2012.

### Study design

The study was implemented as a longitudinal observational study to assess the ability to achieve high coverage within the target population of the use of a uterotonic immediately following birth. The intervention involved activities at the health facility, community and household levels by MOHSW staff and unpaid community volunteers.

Program implementation was preceded by a design phase wherein the Ministry carefully considered the benefits and limitations of a specific intervention to reduce postpartum hemorrhage at home birth. The research questions were: 1) if ANC visits were a feasible and effective mechanism for providing birth preparedness and complication readiness (BP/CR) counseling and distributing misoprostol during pregnancy for PPH prevention to women who deliver at home; 2) whether trained District Reproductive Health Supervisors (DRHSs) could effectively provide counseling on BP/CR and distribute misoprostol to women during home visits; 3) whether the two strategies together could achieve high coverage of uterotonic use for PPH prevention either at facility or home births; 4) whether misoprostol use would be acceptable to women for PPH prevention; and 5) whether advance distribution of misoprostol would affect facility birth rates.

Preparatory activities took place from August to November 2012, and training was conducted in December 2012. Counseling by health workers and distribution of misoprostol started in December 2012 and continued for seven months through June 2013. Approximately 1826 pregnant women age 15–49 were expected to be enrolled, based on the national crude birth rate of 45.0 births per 1000 population, the total population for the two districts and the seven-month period of observation. It was expected that the majority of women would be reached through ANC, and that the remainder would be identified through community mapping by trained traditional midwives (TTMs) and targeted for home visits by the DRHSs. TTMs were trained to identify and educate pregnant woman about misoprostol, but not to distribute the drug.

### The intervention

The PPH prevention program was designed by the MOHSW with the technical assistance of Jhpiego through the Maternal and Child Health Integrated Program (MCHIP). It was implemented by the Grand Bassa County Health Team in Liberia, with technical guidance from Family Health Division of the MOHSW and MCHIP staff. As misoprostol was not available in Liberia, Venture Strategies Innovations provided the misoprostol and ensured its appropriate importation and entry into the regular national commodity distribution system of the Liberian National Drug Service (NDS). Based on evidence from other PPH prevention studies, a dose of 600 mcg (three tablets, each 200 mcg) misoprostol tablets taken orally was chosen.

Facility-based care at eight of nine health facilities was strengthened by training the providers who offer ANC, delivery services and postnatal care. Health care providers who attend deliveries were trained on AMTSL, especially the administration of oxytocin for the prevention of PPH, and the management of PPH among women who took with misoprostol at home. Training used competency-based methods, and all providers were competent at the conclusion of the training, as measured by objective skill assessment tools. For the advance distribution intervention, health care workers at facilities were trained to provide the following during ANC: provision of specific messages on BP/CR and the risks of PPH; identification and consenting of eligible women; counseling on the use of misoprostol; and advance distribution of misoprostol to women at or after 32 weeks of pregnancy for self-administration following home birth.

For prevention of PPH at home birth, DRHSs were trained in counseling and advance distribution of misoprostol. DRHSs are certified/registered midwives who work at hospitals and health clinics and conduct outreach in communities and women’s homes. They supervise other staff, including TTMs. DRHSs were trained to identify, with the help of the TTMs, women who had not completed the recommended ANC visits, to obtain oral consent, and to counsel them on BP/CR and the importance of facility-based delivery. If the woman was at eight months of pregnancy, the DRHS would provide a dose of misoprostol for the woman/family to hold until the time of delivery, with instructions to take the medication immediately following the birth of the baby (with confirmation of no undiagnosed second twin) and before the delivery of the placenta. TTMs, MOHSW-recognized volunteer CHWs, were trained in community mapping to identify all pregnant women in their community and to hold community sensitization activities. TTMs counseled women late in pregnancy using pictorial cards on the importance of ANC, birth preparedness, facility-based birth and appropriate preparation for birth; the danger signs of pregnancy, especially PPH; and the use of misoprostol for PPH prevention. TTMs did not distribute the misoprostol, but reinforced the messages about misoprostol obtained either from the clinics or the DRHS.

All participants were trained in record keeping and data recording, using specific data collection tools designed according to their corresponding role.

Counseling by all providers was facilitated by specifically designed, nationally appropriate pictorial message cards. The educational messages to women regarding misoprostol were to: store it in a safe place until the time of delivery; never take the medication prior to the birth of the baby; ensure that the TTM check for the presence of a second baby immediately following delivery at home; and swallow the three tablets with water immediately after the delivery of the baby and before the delivery of the placenta. Women were advised about transient side effects and the possibility of continued bleeding, even if the medication was taken correctly. In the face of persistent side effects or continued bleeding, women were advised to go to a facility. If women chose delivery in a facility, they were instructed to bring their misoprostol, which would be returned to the provider who would destroy it and administer oxytocin. Women who consumed the medication at home were instructed to bring the empty blister packets with them to the health facility during their postpartum visit and newborn’s first immunization visit.

Misoprostol was obtained from Cipla Pharmaceuticals (India) in four-tablet strips. Once the medication arrived in country and was entered into the NDS system, it was repackaged by the county pharmaceutical system before distribution to the health facilities and DRHSs. Repackaging involved cutting the four-tablet strips into three-tablet strips and placing them in a small sachet with an instruction card for use.

### Exclusion criteria

Pregnant women who consented and met all of the following eligibility criteria were provided with misoprostol: aged 15 years or older; had reached 32 weeks gestation or greater; had no known history of allergy to prostaglandins; and had not previously had a cesarean section. Women who had or developed a chronic disease over the course of her pregnancy (such as cardiac disease, diabetes, pregnancy-related hypertension or any other high-risk condition) were not eligible to receive misoprostol. These women were strongly encouraged to deliver in a facility and informed about risks involved in delivery without skilled attendance.

### Data collection and analysis

Data on misoprostol distribution were collected at health facilities using modified ANC registers and at home by DRHSs using a Community Register for Recording Counseling and a Misoprostol Distribution and Delivery Information register. Data were tallied monthly in Monthly Misoprostol Consumption Log Books. Data on facility-based deliveries and any complications were extracted from the Labor and Delivery Registers. Data on home births were reported by TTMs to the health facility and others were captured during postpartum or first immunization visits. If women experienced a severe adverse event or were referred to another facility due to severe complications, providers recorded data using the Health Facility Admission Form. Baseline and prospective data on ANC visits and numbers of deliveries at the facility were collected from routine reporting into the Health Management Information System (HMIS).

Among the women who were enrolled and received advance distribution of misoprostol, 550 were randomly selected from the ANC and DRHS registers for a follow-up interview postpartum. The sample was arrived by using Epi Info 7 StatCalc using estimated PPH prevalence at 20% at 95% confidence interval and design effect of 2. Using this calculation, 500 interviews were required, and 10% additional interviews were added to account for no-responses. The group that received misoprostol from DRHS was oversampled to allow for better understanding of the role that cadre played in the education of women and provision of misoprostol and messages. Postpartum interviews were intended to be conducted by the DRHSs, but it was determined not to be feasible during implementation due to the small number of DRHS and their broad array of responsibilities. MCHIP hired dedicated interviewers and trained them to go to the houses of the selected women to administer the questionnaire.

All data collection forms were manually reviewed for completion and accuracy by the MCHIP office in Liberia. The MCHIP Liberia and Washington teams assisted with data entry and analysis using Microsoft Access. Initial analysis was performed in Washington, DC using Stata 10.0 (Stata Corp, College Station, TX, USA). A trend analysis was conducted on AMTSL data to examine the proportion of women with vaginal births receiving a uterotonic, as well as the numbers of ANC visits and births at facilities from baseline to the end of the program. The misoprostol distribution analysis included summary statistics of distribution and cross-tabulation of key outcome indicators. Results were summarized using frequency distributions and cross tabulations.

## Results and discussion

### Misoprostol distribution and coverage

**Figure 1:**
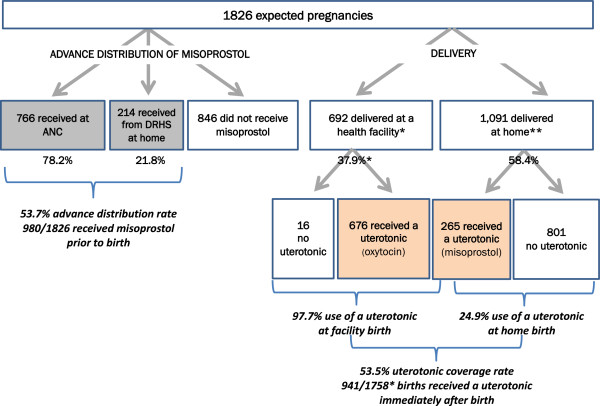
**Distribution and uterotonic coverage rates of expected deliveries in intervention qrea (December 2012–June 2013).** *Recorded vaginal deliveries only, excluding the 68 cesarean deliveries (account for 3.7% of expected deliveries) because provision of uterotonic tothose women is unclear. **Total expected number of home deliveries (calculated as 1758–692 recorded facility deliveries).

During the seven months of implementation, 1,826 deliveries were expected in the intervention area. Figure 1 presents data on misoprostol distribution rate by source. A total of 980 (53.7%) of pregnant women were contacted and enrolled in the program. Of these, 766 (78.2%) were reached at the health facilities during ANC visits, and 214 (21.8%) were reached at home by a DRHS. An estimated 46.3% of women did not receive advance distribution of misoprostol with counseling.Figure 1 also presents data on uterotonic coverage for facility-based and home births. A total of 692 vaginal births at facilities were recorded. The national clinical protocols do not explicitly state whether a uterotonic should be used for cesarean section deliveries, and uterotonic use was not recorded for these deliveries (n = 68). They therefore have been excluded from coverage calculations.

Among 692 facility-based deliveries, 676 (97.7%) women received oxytocin, as collected through the project registers at the health facilities. Before this intervention, policy allowed only skilled birth attendants to provide a uterotonic, which was recorded on the partograph or the woman’s hospital record. This indicator currently is not captured in the country’s HMIS.

Among the remainder of estimated deliveries were expected to have occurred at home (n = 1066), 265 (24.9%) women took misoprostol for self-administration after home birth. The overall uterotonic coverage rate for both facility and home births was 53.5%.

### ANC Visits and deliveries at health facilities

**Figure 2:**
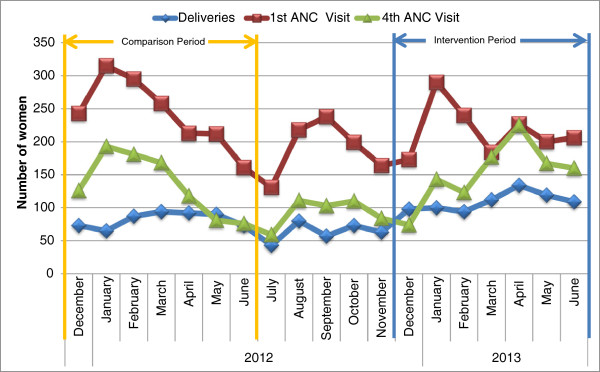
**Number of deliveries in participating facilities (Dec 2011–June 2013).** Blue line represents the number of deliveries. Red line represents the number of ANC1 visits**.** Green line represents the number of ANC4 visits.

Data on ANC1 visits, ANC4 visits and deliveries in participating health facilities were collected from the national HMIS for the period December 2011–June 2013. (Figure 2) The monthly average of ANC1 and ANC4 visits during the intervention period appears to be essentially unchanged when compared to the same period in the previous year. The average monthly number of facility deliveries, however, increased from the 82 during the comparison period (December 2011–June 2012) to 108 during the intervention period (December 2012–June 2013).

### Use of misoprostol for home births

From a sample of 550 women who received advance distribution of misoprostol and were interviewed postpartum, a large number of women (45.1%) who were provided misoprostol came to a facility for their delivery (Table 2). Per protocol, the facility staff destroyed the tablets and administered oxytocin. Of the 302 women who reported they delivered at home, 265 women (87.7%) self-administered misoprostol.

**Table 2:** Place of birth and misoprostol use among enrolled women interviewed postpartum (n = 550)

**Indicator**	**Number**	**Percentage**
**Reported place of delivery for women (n = 550):**	**550**	
Health facility	248	45.1%
Home	302	54.9%
**Reported misoprostol use (n = 302):**	**302**	
Home births and took misoprostol	265	87.7%
Home births and did NOT take misoprostol	37	12.3%

Women who self-administered misoprostol were asked about timing of ingestion, and 167 (63.0%) reported they took the drug at the correct time, and 87 (32.8%) incorrectly took the drug after the delivery of the placenta (Table 3). Three women (1.1%) reported taking the drug prior to the delivery of the baby. Their reasons are unclear, but each woman, when interviewed, reported no negative effect. Women counseled by ANC providers were more likely to ingest misoprostol at the correct time (70.5%) compared to those counseled by a DRHS (50.5%) (p-value = .002).

**Table 3:** Correct and incorrect use of misoprostol, by source of counseling, among enrolled women interviewed postpartum who received advance distribution of misoprostol, had a home birth and reported using the drug (n = 265)

**Indicator**	**Total (n = 265)**	**Health care providers during ANC (n = 166)**	**DRHSs during home visits (n = 99)**
Misoprostol taken CORRECTLY (immediately after delivery of baby AND before delivery of placenta)	167 (63.0%)	117 (70.5%)	50 (50.5%)
Misoprostol taken INCORRECTLY (BEFORE delivery of baby)	3 (1.1%)	2 (1.2%)	1 (1.0%)
Misoprostol taken INCORRECTLY (AFTER delivery of baby and placenta)	87 (32.8%)	45 (27.1%)	42 (42.4%)
No response (data not available)	8 (3.0%)	2 (1.2%)	6 (6.1%)

### Birth planning and knowledge about PPH and misoprostol

Women interviewed postpartum who were enrolled in the intervention were asked questions to assess their recall of key counseling messages and planning for delivery. Over 90% of women correctly answered questions about misoprostol (Table 4). Recall of at least one danger sign was lower, but greater than 80%. Around half of the women interviewed had made some arrangements for transportation for delivery, and slightly higher number of women had saved money for an emergency. Results were similar whether women were counseled by a health care provider or by DRHS.

**Table 4:** Birth planning and knowledge, by source of counseling, among enrolled women interviewed postpartum (n = 550)

**Indicator**	**Health care providers during ANC (n = 401)**	**DRHSs during home visits (n = 149)**	**p-value**
**Knowledge:**			
Danger signs during pregnancy (at least one)	339 (84.5%)	125 (83.9%)	0.799
Signs of excessive bleeding	328 (81.8%)	136 (91.3%)	0.013
Purpose of misoprostol to reduce bleeding	383 (95.5%)	147 (98.7%)	0.457
Timing of self-administration	383 (95.5%)	147 (98.7%)	0.003
Number of pills to take	384 (95.8%)	147 (98.7%)	0.308
Side effects of misoprostol (at least three side effects)	293 (73.1%)	138 (92.6%)	0.000
**Birth planning:**			
Arranged for transport during delivery	182 (45.4%)	81 (54.4%)	0.106
Saved money for delivery	264 (65.8%)	103 (69.1%)	0.148

### Women’s satisfaction with misoprostol

Satisfaction was measured by three questions in the postpartum interview. A very high proportion of women who reported giving birth at home and taking misoprostol (n = 265) reported being satisfied with their use of the medication (Table 5).

**Table 5:** Women’s reported satisfaction, by source of counseling, among enrolled women interviewed postpartum who used misoprostol (n = 265)

**Indicator**	**Health care workers during ANC (n = 166)**	DRHSs during home visits (n = 99*)	**p-value**
**Women who were satisfied with use of misoprostol:**			
Would recommend misoprostol to a friend (n = 259)	165* (100.0%)	93 (98.9%)	0.403
Agreed to pay 5 Liberian Dollars** (n = 260)	92 (55.4%)	50 (53.2%)	0.762
Would take misoprostol for next delivery (n = 259)	162* (98.2%)	92 (97.9%)	0.937

*Note: Some women did not answer all questions, therefore the denominator is not always based on ‘n’.

**Approximately $.06 USD.

### Side effects, adverse events, obstetric complications and referrals

The women who used misoprostol during home births (n = 265) were asked during the postpartum interview if they experienced any minor side effects, adverse events or complications. A total of 143 women (54.0%) indicated that they experienced at least one minor side effect including dizziness, nausea, shivering, vomiting, fever, abdominal cramping or watery stools. The majority reported that the side effects subsided after 30–60 minutes. There were no severe adverse events (defined as uterine rupture, fever more than 40 degrees, or death of a woman enrolled in the intervention) reported. Of the 232 misoprostol users who were multigravida, 191 women (82.3%) stated that they bled less than previous birth experiences. Four women were referred for retained placenta or bleeding.

### Health care providers’ and DRHSs’ knowledge of misoprostol

Health care providers (9) and DRHSs (2) trained in PPH interventions were asked questions to measure their knowledge about misoprostol as a follow-up to the training. All (100%) knew when the woman should self-administer misoprostol, and ten (90.9%) correctly answered how misoprostol works.

### Availability of oxytocin and misoprostol at distribution points

During the intervention one clinic reported stock-out of misoprostol during one month, due to transportation problems. There were no recorded stock-outs of oxytocin from any of the facilities.

## Discussion

In the low-resource and post-conflict setting of Liberia, the dual strategies for provision of a PPH prevention intervention were able to ensure provision of a uterotonic for 53.5% of all estimated vaginal deliveries (facility and home births combined). The intervention found that it is safe to provide women with a dose of misoprostol in advance of their delivery for self-administration after the birth should they deliver at home. There were no adverse events reported, including among the three women who took misoprostol prior to the birth of the baby. This is consistent with the review that found self-administration of misoprostol programs appears to be safe, with an extremely low rate (.06%) of incorrect antepartum or intrapartum administration [12]. It however is concerning that a substantial proportion (33%) of women incorrectly took the medication after the delivery of the placenta. While women’s knowledge on PPH and misoprostol were high, mistimed self-administration indicates a need for strengthened counseling during ANC and home visits.

When women delivered at home and had a dose of misoprostol, 88% of them reported taking it. This demonstrates that when women have the drug in hand at a home birth, they are willing to take it. Misoprostol appears acceptable to pregnant women for PPH prevention. About 98% of the women who took misoprostol said that if they become pregnant again they will take the drug and would recommend the drug to a friend or relative. Only about 50% of these women were willing to pay for it, due perhaps to expectations that medicines are provided free of cost per MOHSW policy.

This program used skilled health care providers, both ANC providers and DRHSs, for distribution. The first distribution strategy through ANC visits achieved the higher distribution rate, as was expected. Of the 1826 anticipated births in the catchment area, 41.9% were enrolled through ANC, less than the overall national ANC4 rate of 66%. Of the 1069 women reported in HMIS who came for ANC visits in late pregnancy, 766 (71.7%) were provided counseling and misoprostol. It is unclear why nearly 30% of women did not enroll during ANC, and this issue needs further exploration during program expansion.

The household-based advance distribution strategy involving DRHSs to reach pregnant women at home was less effective. Of the 1826 anticipated births in the catchment area, 11.7% were enrolled through home visits. Of the total number of women who delivered at home, only 24.3% were provided with a uterotonic. By design, the TTMs identified pregnant women who were not attending ANC and informed the DRHS to visit them at home. The low misoprostol distribution rate by DRHS may be due to the small number of mobilized DRHSs for distribution, logistical constraints, hard to reach communities, large catchment area and other facility-based responsibilities of DRHSs.

These two distribution strategies rely on the health system (ANC providers and DRHSs) and did not result in high uterotonic coverage. Global evidence indicates that greatest distribution and coverage are achieved by CHWs during home visits [12]. Using TTMs or other existing CHWs may be considered as an advance distribution strategy to increase the coverage of women who are unable to attend ANC due to poor geographic access and likely to deliver at home. In Liberia, TTMs provide support and are a source of knowledge related to pregnancy and maternal health in the community [15]. Engaging the TTMs more actively and allowing them to distribute misoprostol as is done in similar settings in other countries, may be necessary to achieve the desired high coverage [16,17]. CHWs may have more contacts, time and trust to provide counseling, especially concerning the correct timing of self-administration of misoprostol. Ultimately, more community engagement and additional innovative strategies may also be needed to reach the most remote and vulnerable women.

For facility-based births, the use of oxytocin immediately following delivery is very high for vaginal births. Records indicate that almost 98% of women who delivered in the facility received oxytocin. While it has been suggested that oxytocin administration in the third stage during labor is routine in Liberia, it is recommended that provision after a cesarean section should be detailed in national protocols. It is promising to note that advance distribution of misoprostol for self-administration at home birth does not appear to reduce the numbers of women coming to deliver at facilities. The number of facility births in the two districts started to increase before the intervention and continued to increase during the intervention. While the increase and the program may be associated, it is not possible from this analysis to suggest causality. It is important, however, to note that facility-based delivery rates did not decrease during the intervention period as noted in other programs [12,18].

Anecdotally the County Health Teams reported seeing fewer cases of PPH during the study, although the study was not designed to assess the clinical effectiveness of misoprostol. The effectiveness of misoprostol in a home birth setting already has been proven to significantly reduce acute PPH and mean blood loss [19].

Commodity availability to avoid stock-outs and appropriate packaging of misoprostol (three tablets) must be addressed during scale-up. As the number of facility-based births increases, a significant amount of misoprostol will be returned unused and destroyed. Use and disposition plans must be developed to prevent wastage, including a consideration of administration of the available misoprostol to women who present to the facility for delivery. HMIS needs to incorporate PPH-related monitoring and evaluation, including an indicator of uterotonic provision.

Liberia has taken bold steps to transition from an emergency relief model of health service delivery to a functioning, decentralized health system. The MOHSW has focused on interventions to improve the quality of service provision at health facility level and has institutionalized a Performance Based Financing (PBF) mechanism. Under PBF, a mechanism for setting performance targets at the central, county, and health facility level is established along with county implementing partners. Counties can either earn or lose a bonus based on whether they meet the targets. This accountability framework and quality improvement efforts at health facility level combined with a strong community focused approach of counseling and misoprostol distribution can be very promising in increasing uterotonic coverage.

### Limitations

This study had numerous limitations. It is not known who benefited most from this intervention and whether those who received the misoprostol were those who were physically close to health facilities already. Additional data are needed about distance to health facilities among those women who accept and use the misoprostol. As well, the community mapping approach may yield more exact figures on the actual number of deliveries in the catchment area, and thus more accurate coverage figures. The study was not designed or powered to assess actual impact of the intervention on reducing PPH incidence or related mortality.

## Conclusions

Efforts to increase uterotonic coverage need to reach and benefit the greatest number of pregnant women, irrespective of the place of delivery (facility or home). This program showed that uterotonic coverage in the third stage of labor was nearly universal (98%) among vaginal births at facilities. Therefore, focus is needed to increase uterotonic coverage for home births through the use of CHWs for advance distribution, given Liberia’s continued high rate of home births. The combined efforts of DRHSs and health care providers at ANC yielded a uterotonic rate of 54%.

### Endnote

^a^At the time the intervention was designed the MMR was 994 maternal deaths per 100,000 live births. More recent data from the 2012 WHO, UNICEF, UNFPA and the World Bank estimates report *Trends in Maternal Mortality 1990–2010* estimate MMR at 770.

## Abbreviations

AMTSL: Active management of the third stage of labor; ANC: Antenatal care; BP/CR: Birth preparedness and complication readiness; CHW: Community health worker; DRHS: District reproductive health supervisors; HMIS: Health management information system; LDHS: Liberia Demographic and Health Survey; MCHIP: Maternal and Child Health Integrated Program; MOHSW: Ministry of Health and Social Welfare; NDS: National Drug Service; PBF: Performance based financing; PPH: Postpartum hemorrhage; TTM: Trained traditional midwife.

## Competing interests

All authors are current employees of the Liberian MOHSW, Jhpiego or JSI. Jhpiego has been involved for many years in implementation of programs to reduce PPH at homebirth using misoprostol throughout Africa and Asia. The authors declare that they have no competing interests.

## Authors’ contributions

JMS, SDB and MS conceived of the study and participated in its design and coordination. VG, MS, GI, BZT and CJH managed implementation of the study. All authors conducted analysis and developed the findings. JMS and VD contributed to the writing of the manuscript. All authors read and approved the final version of the manuscript.

## Pre-publication history

The pre-publication history for this paper can be accessed here:

http://www.biomedcentral.com/1471-2393/14/189/prepub
